# Alexithymia: a general deficit of interoception

**DOI:** 10.1098/rsos.150664

**Published:** 2016-10-12

**Authors:** Rebecca Brewer, Richard Cook, Geoffrey Bird

**Affiliations:** 1 MRC Social, Genetic and Developmental Psychiatry Centre, Institute of Psychiatry, King's College London, London SE5 8AF, UK; 2 School of Psychology, University of East London, University Way, London E16 2RD; 3 Department of Psychology, City University London, London EC1V OHB, UK; 4 Institute of Cognitive Neuroscience, University College London, London WC1N 3AR, UK

**Keywords:** alexithymia, emotion, interoception

## Abstract

Alexithymia is a sub-clinical construct, traditionally characterized by difficulties identifying and describing one's own emotions. Despite the clear need for interoception (interpreting physical signals from the body) when identifying one's own emotions, little research has focused on the selectivity of this impairment. While it was originally assumed that the interoceptive deficit in alexithymia is specific to emotion, recent evidence suggests that alexithymia may also be associated with difficulties perceiving some non-affective interoceptive signals, such as one's heart rate. It is therefore possible that the impairment experienced by those with alexithymia is common to all aspects of interoception, such as interpreting signals of hunger, arousal, proprioception, tiredness and temperature. In order to determine whether alexithymia is associated with selectively impaired affective interoception, or general interoceptive impairment, we investigated the association between alexithymia and self-reported non-affective interoceptive ability, and the extent to which individuals perceive similarity between affective and non-affective states (both measured using questionnaires developed for the purpose of the current study), in both typical individuals (*n* = 105 (89 female), mean age = 27.5 years) and individuals reporting a diagnosis of a psychiatric condition (*n* = 103 (83 female), mean age = 31.3 years). Findings indicated that alexithymia was associated with poor non-affective interoception and increased perceived similarity between affective and non-affective states, in both the typical and clinical populations. We therefore suggest that rather than being specifically associated with affective impairment, alexithymia is better characterized by a general failure of interoception.

## 1. Background

Research on alexithymia, traditionally defined in terms of difficulties identifying and describing one's own emotions [1], has focused relatively little on the ability to perceive non-emotional states from the body (termed ‘interoception’). Interoception refers to the perception of a wide range of physical states beyond emotions, including heart rate, respiratory effort, temperature, fatigue, hunger, thirst, satiety, muscle ache, pain and itch [2–4]. This description is given anatomical specificity through the definition of interoception as the processing of any bodily information that is sent via lamina I to the insula or anterior cingulate cortex (ACC) [2], or via cranial nerves to the nucleus of the solitary tract [5]. The ability to interocept has recently been further qualified, by separation into three constituent parts [6]. Under this three-dimensional model, interoceptive sensitivity refers to the objective accuracy of one's interoception (e.g. ability to perceive accurately one's heart rate). Interoceptive sensibility, on the other hand, assesses one's subjectively perceived sensitivity (the extent to which one believes one is accurate at perceiving bodily states), as well as beliefs about the state of one's body. Finally, interoceptive awareness is defined as a metacognitive awareness of interoceptive ability (the extent to which one's subjective interoceptive sensibility accurately reflects one's objective interoceptive sensitivity).

At the neural level, the regions most commonly implicated in interoception are the anterior insula (AI) and the ACC, which are highly involved in processing non-affective interoceptive states [2–4,6], and often referred to as the ‘interoceptive cortex’. Notably, these brain regions are not only involved in non-affective interoception; they also play a central role in processing one's own emotions [7–11]. Indeed, the same insular region has been shown to be active when individuals perform affective and non-affective interoceptive tasks [12]. At the neural level, therefore, there appears to be a high degree of overlap between non-affective and affective interoception.

Interestingly, alexithymia is commonly associated with abnormalities of the AI and ACC, with atypical structure and function having been observed in both regions [13–15]. As emotional processing and interoception appear to rely on the same neurological system, it follows that alexithymia (associated with atypicalities in interoceptive cortex) should be associated not only with difficulties identifying one's own emotions, but also with identifying one's non-affective internal states. It is possible that those who struggle to identify whether they are feeling anger or fear, for example, may also have difficulty identifying and differentiating states such as hunger, arousal and fatigue.

Support for the hypothesis that individuals with alexithymia atypically represent non-affective, as well as affective, interoceptive states is mounting. Those with alexithymia are less accurate than typical individuals in perceiving their own heart rate [16,17], for example, and are delayed in seeking medical treatment in response to acute myocardial infarction [18]. Alexithymia is also associated with erratic consumption of caffeine [19], alcohol [20] and other substances [21,22]. Increased use of these substances may be a consequence of decreased awareness, or misinterpretation, of their effect on the body. Those with alexithymia also exhibit atypical activation of the insula relative to those without alexithymia in response to colonic distension (stimulation of the colon through inflation of a barostat bag) [23].

Notably, alexithymia frequently co-occurs with clinical disorders that are associated with poor interoception. Levels of alexithymia are elevated, for example, in individuals with feeding and eating disorders (EDs) [24,25], which are characterized by decreased interoceptive ability [26,27] relating to reduced perception of signals of hunger and satiety. Clinical levels of substance abuse are associated with increased alexithymia severity [28], as well as with poor awareness of intoxication levels and reduced sensitivity to reward signals [29] (markers of interoceptive sensitivity). Alexithymia also frequently co-occurs with autism spectrum disorder (ASD), and these individuals may respond atypically to affective touch, which relies upon interoceptive, rather than exteroceptive neurobiological systems [30]. Importantly, atypicalities in processing ‘interoceptive’ touch may relate specifically to the variety of touch atypicalities exhibited by individuals with ASD, such as dislike of physical contact, and sensitivity to particular fabrics [31,32]. Finally, high levels of alexithymia have been observed in diabetes, which is characterized by difficulty regulating blood glucose levels in order to maintain homeostasis [33–35], as well as in numerous other medical conditions [36]. It therefore seems likely that alexithymia is not purely associated with deficits relating to the identification of one's emotions (affective interoception), but instead with interoceptive failure more generally. Conclusive evidence is lacking, however, as studies that have explicitly investigated the relationship between alexithymia and interoceptive sensitivity have relied solely on measures of heart beat perception [16,17,37]. While this is clearly a useful technique, it does not allow assessment of abilities across interoceptive domains.

If those with alexithymia struggle to perceive and identify interoceptive states and emotions, they may also confuse affective and non-affective interoceptive states with each other. Indeed, the original definition of alexithymia included difficulties distinguishing emotions from other bodily sensations, which was initially observed in psychosomatic patients [1,38]. Although it is typical to associate certain emotions with non-affective interoceptive states (such as feeling hot while experiencing anger [39], or experiencing nausea while feeling disgusted [40]), it is possible that individuals with alexithymia, due to interoceptive confusion, perceive these states as more similar than is typical, or perceive similarity between states that are not typically associated.

The current study investigated the possibility that alexithymia is associated not only with emotion identification and recognition deficits, but also with general interoceptive impairment. This hypothesis was investigated using a self-report method in individuals reporting a psychiatric diagnosis, as well as in the typical population, while taking psychopathy into account due to some overlap between the alexithymia and psychopathy constructs. It should be noted that, while alexithymia co-occurs with a number of disorders, such as ASD and depression, previous research demonstrates that these are distinct constructs [41–43]. Research shows, for example, that emotional difficulties in those with ASD are not due to ASD itself, and that a high proportion of individuals with ASD do not experience these impairments [44,45]. While it is likely that psychopathy and alexithymia are also distinct [46], these constructs have a number of similarities, such as atypical empathy, which is a defining feature of psychopathy [47,48]. Individuals' perceived interoceptive abilities were assessed using a novel self-report questionnaire (the Interoceptive Confusion Questionnaire). Self-report measures assessing interoception, such as the Body Perception Questionnaire (BPQ) [49], the Multidimensional Assessment of Interoceptive Awareness (MAIA) [50] and the Body Consciousness Questionnaire (BCQ) [51], have been used previously. These have been limited, however, by confounding subjective perception of interoceptive sensitivity with the extent to which individuals experience bodily states (BPQ), or by assessing multiple factors, but including a very small number of items assessing interoceptive sensibility (MAIA and BCQ). The current study therefore used a 20-item self-report measure specifically assessing perceived ability to detect interoceptive states (interoceptive sensibility).

## 2. Material and methods

In total, 208 individuals (172 female) between the ages of 18 and 69 years (*M* = 29.5, s.d. = 11.8) participated in the current study. A website (troublewithfaces.com), publicized on social media sites such as Facebook and Twitter, was used to recruit participants and collect data. Participants were first required to report their lifetime history of clinical diagnoses (previous or current). One hundred and five participants had never received a clinical diagnosis, while others reported a current or previous diagnosis of anxiety or depression (73 participants), ASD (seven participants), EDs (12 participants) or another disorder besides those listed (excluding bipolar disorder or schizophrenia as these were available options; 11 participants). It should be noted that the clinical categorizations reported here were derived from participants' reports, not from clinical diagnoses or interviews. Anxiety and depression were included as a single category due to the high degree of co-occurrence between these two conditions.

All participants completed four self-report questionnaires. The Toronto Alexithymia Scale (TAS-20) [52] measured alexithymia severity, and psychopathy was assessed using the Levenson Self-Report Psychopathy Scale (SRPS) [53]. Two self-report measures were created in order to measure interoceptive sensibility. The first of these, the Interoceptive Confusion Questionnaire, assessed the degree to which individuals feel that they struggle to interpret their own non-affective interoceptive states, such as hunger, temperature and arousal. Items from the Interoceptive Confusion Scale are presented below. All items are responded to on a scale from 1 (Does not describe me) to 5 (Describes me very well). Items marked with * are reverse scored (5 = 1, 4 = 2, 2 = 4, 1 = 5). An interoceptive confusion score, ranging between 20 and 100, was calculated by summing all responses following reverse scoring.

### 2.1. Interoceptive Confusion Questionnaire items

1. When I adjust the heat of a room or car, others find it uncomfortable.

2. I frequently forget to eat.

3. I cannot tell when my muscles are sore or tight.

4. When injuries or bruises appear on my body I always know the cause.*

5. I find it hard to know how firmly to hug people.

6. I am quite clumsy and uncoordinated.

7. I frequently eat so much I feel uncomfortable.

8. I don't know whether I'm ticklish or not.

9. When I am feeling anxious, I notice very quickly.*

10. I often find that I'm suddenly very thirsty.

11. It's hard to say whether I will be bored by a task.

12. I am very sensitive to changes in my heart-rate.*

13. I often stay up very late having lost track of time.

14. I always know when I am about to vomit.*

15. I only realize I am stressed when others tell me.

16. I can always tell whether clothes will be irritating on my skin.*

17. Caffeine does not always affect me in the same way.

18. I can tell when I am in love from the way I feel inside.*

19. I can easily tell whether I'm enjoying physical affection.*

20. When lifting objects, I often misjudge their weight.

The second interoception measure, the State–Emotion Similarity Questionnaire, assessed the extent to which individuals experience emotions and non-affective states as similar to each other. Seventy-two items assessed similarity between the six basic emotions and 12 non-affective states. All items were presented in the same format; ‘How similar are your personal experiences of (a state) and feeling (an emotion)?’ and rated on a scale from 1 (not at all similar) to 7 (very similar). The 12 states assessed were feeling hot, feeling cold, nausea, hunger, thirst, physical aches and pains, physical fatigue, shortness of breath, muscle cramp/tension, physical numbness, skin tingling/irritation and racing heartbeat. The six emotions assessed were happiness, sadness, disgust, anger, fear and surprise. Total state–emotion similarity score, ranging from 72 to 504, was calculated by summing all responses.

### 2.2. Data analysis

Correlations were assessed between alexithymia and scores on the Interoceptive Confusion Questionnaire, in the full sample, as well as in the typical and clinical groups. The strength and slope of the relationship between alexithymia and interoceptive confusion were compared between the typical group and the clinical group using Fisher r-to-z tests and Potthoff analyses, respectively.

Identical analyses were conducted for the state–emotion similarity score. In order to determine whether the pattern of associations between states and emotions differed between the alexithymia and control groups, a 3-way emotion (6 levels) × state (12 levels) × alexithymia group (2 levels) ANOVA was used, in which emotion and state were within subject variables, and a single value was included for every possible combination of the 6 states and the 12 emotions, for each participant. This value corresponded to the single rating a given participant gave for the degree of similarity between each state and emotion pair. A significant three-way interaction would indicate that the pattern of state–emotion associations differed between those with and without alexithymia. On the other hand, if alexithymia did not interact with the state × emotion interaction, this would indicate that the pattern of state–emotion associations did not differ significantly between alexithymic and non-alexithymic groups.

## 3. Results

Prior to conducting analyses investigating the relationship with alexithymia, validity and reliability analyses were conducted for both questionnaires in a larger sample of participants. Exploratory factor analysis of the Interoceptive Confusion Questionnaire in a sample of 653 participants indicated an unclear two factor solution, with a low Chronbach's alpha (0.53), despite good test–retest reliability over 12 months (*r* = 0.590, *p* < 0.001). As a consequence, although there was a highly significant positive correlation between alexithymia and interoceptive confusion (*r* = 0.687, *p* < 0.001), and between total state–emotion similarity score and interoceptive confusion (*r* = 0.382, *p* < 0.001), these findings should be interpreted with caution. All further analyses focus on the State–Emotion Similarity Questionnaire.

Exploratory factor analysis of the state–emotion similarity scale, on 290 participants, indicated a one factor solution, with the first factor having an Eigenvalue of 20.73, and explaining 29% of the variance. All 72 items loaded onto this factor, with loadings ranging between 0.32 and 0.68. Cronbach's alpha was excellent, at 0.96, indicating high internal consistency. Analysis on a subset of 36 individuals who completed the questionnaire twice (approx. 12 months apart) indicated high test–retest reliability (*r* = 0.629, *p* < 0.001).

**Figure 1.**
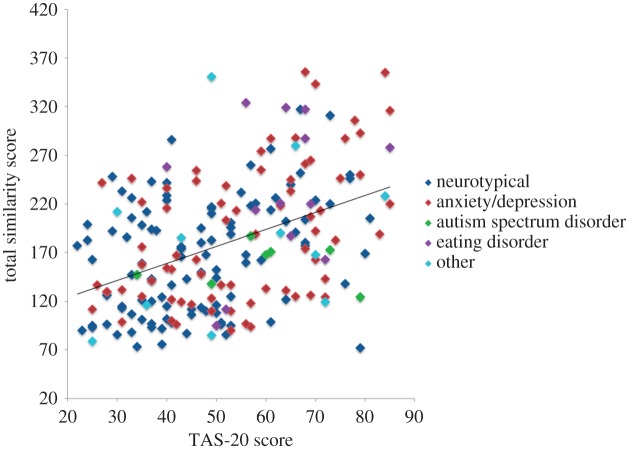
Scatter plot showing significant positive correlation between alexithymia and State–Emotion Similarity score across typical individuals and those reporting clinical diagnoses.

Alexithymia was significantly positively correlated with total perceived similarity between affective and non-affective states (*r* = 0.425, *p* < 0.001; figure 1). Despite alexithymia being significantly correlated with psychopathy (*r* = 0.440, *p* < 0.001), the correlation between alexithymia and total state–emotion similarity score remained once psychopathy was controlled for (*r* = 0.350, *p* < 0.001). Alexithymia predicted state–emotion similarity in the typical group (*r* = 0.343, *p* < 0.001), as well as in those reporting a psychiatric diagnosis (*r* = 0.430, *p* < 0.001), and Fisher r-to-z analyses indicated that the strength of the relationship did not differ between the typical and clinical groups (*z* = 0.725, *p* = 0.468). The slope of the relationship, however, differed significantly between the typical (*β* = 1.352) and clinical (*β* = 1.897) groups (*F*_2,204_ = 3.88, *p* = 0.020).

**Figure 2.**
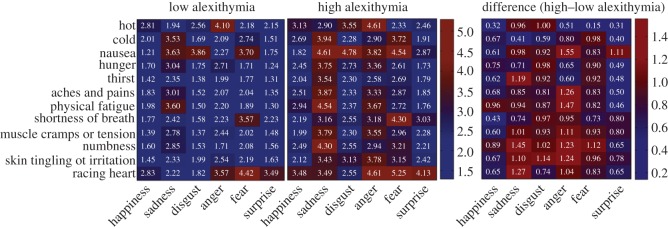
Similarity matrices demonstrating greater perceived similarity between individual emotions and interoceptive states in individuals reporting high levels of alexithymia than in individuals reporting low levels of alexithymia separately, and the difference between these groups.

A significant main effect of alexithymia in an emotion × state × alexithymia group ANOVA suggested that individuals with alexithymia (*M* = 3.05, s.e. = 0.10) rated affective and non-affective states as more similar to each other than did those without alexithymia (*M* = 2.22, s.e. = 0.07) [*F*_1, 206_ = 45.04, *p* < 0.001] (figure 2; see the electronic supplementary material, table S1 for alexithymia group differences for individual state–emotion pairs). While the alexithymia group interacted significantly with emotion (*F*_5, 1030_ = 2.34, *p* = 0.040) and state (*F*_11, 2266_ = 2.38, *p* = 0.006), a non-significant emotion × state × alexithymia group interaction indicated no difference in the relationship between specific states and specific emotions in individuals with and without alexithymia. Although the qualitative pattern of similarities differed numerically between the groups, therefore, it was not significantly different; individuals with alexithymia simply showed typical associations (such as associations between anger and feeling hot [39], and disgust and feeling nauseated [40]) to a greater extent. Main effects of emotion (*F*_5, 1030_ = 88.06, *p* < 0.001) and state (*F*_11, 2266_ = 67.07, *p* < 0.001) indicated that emotions and states vary in the extent to which they are perceived to overlap with states and emotions, respectively. Perceived similarity between affective and non-affective states was not affected by gender (*t*_205_ = 0.829, *p* = 0.408).

## 4. Discussion

The current study investigated whether alexithymia, a condition traditionally defined by affective interoception impairment (difficulties interpreting one's own emotion) is characterized better by a general failure of interoception, in line with evidence suggesting that alexithymia is associated with reduced awareness of one's heart beats [16,17], and increased consumption of caffeine [19], alcohol [20] and other substances [21,22]. Using self-report measures, the association between alexithymia and non-affective interoceptive abilities was investigated in typical individuals, and in those with a self-reported psychiatric condition. Findings suggested that, in both the typical and clinical populations, increased levels of alexithymia were related to a greater degree of perceived similarity between affective and non-affective interoceptive states (as well as increased reporting of interoceptive confusion, although the poor psychometric properties of this measure impede interpretation of this finding). Those with alexithymia therefore not only struggle to recognize their own emotions (affective interoception) but also confuse these states with non-affective interoceptive states, such as hunger, tiredness and arousal. Alexithymia may, therefore, be characterized better by a general failure of interoception, rather than one of affective interoception alone.

The current findings imply that deficits experienced by those with alexithymia are broader than currently acknowledged [54]. Clearly, misinterpreting anger as heat, pain or hunger (or vice versa), for example, is likely to have a wide variety of negative consequences for one's physical and mental health, through reduced ability to respond appropriately to the state and reduce any negative arousal. Social consequences for individuals with alexithymia may also be more severe than originally thought; it is possible that individuals with alexithymia struggle to identify not only emotion in others [55–57], but also signals of non-affective interoceptive states, such as heat, nausea and hunger. The ability to recognize these states is particularly important in order to respond to others' needs, so if it is, indeed, the case that alexithymia impairs this ability, it is likely to negatively impact on one's ability to successfully care for others. The current results also highlight the clinical significance of alexithymia in psychiatric and neurological disorders characterized by elevated levels of alexithymia. These include ASD, EDs, post-traumatic stress disorder and substance abuse, as well as conditions such as diabetes, human immunodeficiency virus (HIV), multiple sclerosis and frontotemporal dementia [24,25,28,34,58–65]. As the current findings suggest that alexithymia may have a similar impact in each of these disorders as it does in the typical population, individuals with these conditions are more likely to suffer interoceptive impairment, due to their elevated risk of alexithymia. Indeed, many of these disorders are already known to be associated with interoceptive deficits, such as recognition of hunger and satiety in EDs, and interpretation of internal reward signals in substance abuse. It is even possible that atypical interpretation of interoceptive signals, such as hunger, satiety and reward cues, plays a causal role in the development and maintenance of these disorders. As it is possible to improve interoceptive sensitivity through training [66], future work should aim to determine the clinical utility of interoceptive training across diagnostic categories. Interventions inducing increased attention to one's internal states may lead to enhanced sensitivity to affective and non-affective interoceptive signals and therefore provide therapeutic benefit across a range of psychiatric conditions. It should be noted, however, that it is necessary for future work to determine whether alexithymia interacts with diagnosis in predicting the ability to interocept using larger samples matched for demographic factors and intellectual functioning. This is particularly relevant as results indicated that the slope of the relationship between alexithymia and perceived similarity between affective and non-affective states differed between the typical and clinical groups. It may be the case, for example, that interoceptive impairment associated with alexithymia is exaggerated in those with EDs, due to deliberate attempts to suppress internal signals of hunger and fatigue in order to restrict eating and exercise excessively. Similarly, replication using in-depth clinical interviews is required in order to investigate the relationship between alexithymia and interoception having accounted for disorder symptom severity.

Should alexithymia be a product of general interoceptive impairment in all of the conditions it is associated with, it is possible that interoceptive impairment may constitute an underlying factor that characterizes a number of distinct developmental, psychiatric and neurological conditions. Indeed, when factor analytic methods are used in order to uncover higher order structure among symptoms from diverse diagnostic categories within psychiatry [67], studies demonstrate the existence of a single higher order factor, the ‘p Factor’, representing lesser-to-greater severity of psychopathology with associated disruption in neural circuitry [68,69]. Given the reliance upon interoception of emotion processing [70–73], learning and decision-making [74], cognitive control [75] and social ability [44,65,76,77], it is possible that interoceptive ability may constitute the p Factor itself. It is possible, for example, that interoceptive impairment may contribute to the development of EDs, due to poor awareness of hunger and satiety cues, substance abuse, due to decreased perception of reward signals, anxiety disorders, due to atypical interpretation of heart rate and respiratory cues, schizophrenia, due to impaired representation of the self as distinct from others (assumed to rely on interoception [78]), and the learning and sensory atypicalities in ASD. This hypothesis is worthy of investigation, as it would represent a significant opportunity for therapeutic intervention, and for aetiological studies, across disorders. Longitudinal studies investigating the relationship between early interoceptive abilities and subsequent disorder diagnosis are likely to aid in testing this hypothesis.

It is worth noting that, while the current study observed decreasing interoceptive sensibility with increasing alexithymia, a recent study using the BPQ observed the opposite relationship [79]. This is at odds with the literature observing decreased interoceptive accuracy in those with alexithymia [16,17], and therefore requires further investigation. It would be beneficial for future research to adapt the Interoceptive Confusion Questionnaire reported here, in order to produce a valid and reliable self-report measure of interoceptive abilities (beyond the perceived State–Emotion Similarity Questionnaire). Furthermore, it is important to determine the relationship between interoceptive sensibility and both objective interoceptive sensitivity and metacognitive awareness. Existing evidence suggests reduced sensitivity in alexithymia [16,17], and the current results suggest decreased sensibility (self-reported impairment). If the interoceptive sensibility measures correlate well with sensitivity, this would imply unimpaired metacognitive awareness in those with alexithymia: that they are able to perceive accurately their interoceptive impairment. It may, however, be the case that individuals with alexithymia are not sensitive to the degree of impairment they face. Further investigation into the nature of the impairment in each of the three interoceptive factors is therefore required. Specifically, future research should compare sensibility and sensitivity across a range of internal states, in order to determine whether the relationship between subjective and objective interoceptive abilities varies across interoceptive domain. It is worth noting that, as two factors were observed in the Interoceptive Confusion Questionnaire reported here, objective tests may observe multiple facets of interoceptive ability.

In conclusion, it seems that the breadth of impairment experienced by individuals with alexithymia is far greater than traditionally thought. The current study extends evidence that alexithymia is associated with affective interoception deficits, and suggests that the condition should be thought of as a general impairment of interoception (including non-affective internal states). Those with alexithymia are likely to confuse non-affective internal states with emotional experiences, and may find it difficult to identify cues relating to states such as hunger, satiety and arousal in themselves. Importantly, the relationship between alexithymia and interoceptive confusion appears to be present across typical and clinical populations. Further research is clearly necessary in order to investigate the (likely complex) impact of alexithymia in different disorders. A clearer understanding of the interoceptive impairment associated with alexithymia, across the three-dimensional construct of interoceptive ability, is also required, using experimental methods. The current findings provide initial evidence, however, that alexithymia is synonymous with interoceptive impairment.

## Supplementary Material

Table S1. Table of t-tests indicating group difference for state-emotion similarities.

## Data accessibility

Data are available as the electronic supplementary material (table S2).
