# Supplementary material for: Alexithymia: a general deficit of interoception
Source: R Soc Open Sci. 2016 Oct 12;3(10):150664. doi: 10.1098/rsos.150664 (PMC5098957; doi:10.1098/rsos.150664)
Supplement: Table S1. Table of t-tests indicating group difference for state-emotion similarities. [file rsos150664supp1.docx]

**Table S1.** Table of *t*-tests indicating group difference for state-emotion similarities. * *p* < .05, ***p* < .01, ****p* < .001. Note that these values are provided for effect size information only, and have not been corrected for multiple comparisons. Following Bonferroni correction, 19 comparisons remain significant.

| State-emotion pair | High alexithymia mean (SD) | Low alexithymia mean (SD) | *t* |
| --- | --- | --- | --- |
| Happiness - hot | 2.81 (1.76) | 3.13 (1.72) | 1.23 |
| Happiness – cold | 2.01 (1.37) | 2.69 (1.84) | 2.66** |
| Happiness – nausea | 1.21 (.69) | 1.82 (1.38) | 3.41** |
| Happiness – hunger | 1.70 (1.14) | 2.45 (1.79) | 3.18** |
| Happiness – thirst | 1.42 (.87) | 2.04 (1.57) | 3.01** |
| Happiness – ache pain | 1.83 (1.28) | 2.51 (1.70) | 2.87** |
| Happiness – physical fatigue | 1.98 (1.38) | 2.94 (1.95) | 3.60** |
| Happiness – shortness of breath | 1.77 (1.29) | 2.19 (1.41) | 2.14* |
| Happiness – muscle cramp/tension | 1.39 (.91) | 1.99 (1.42) | 3.14** |
| Happiness – numbness | 1.60 (1.12) | 2.49 (1.76) | 3.79*** |
| Happiness – skin tingling/irritation | 1.45 (.91) | 2.12 (1.59) | 3.18** |
| Happiness – racing heart | 2.83 (1.80) | 3.48 (1.80) | 2.39* |
| Sadness - hot | 1.94 (1.37) | 2.90 (1.98) | 3.56** |
| Sadness – cold | 3.53 (1.92) | 3.94 (1.92) | 1.51 |
| Sadness – nausea | 3.63 (2.13) | 4.61 (1.94) | 3.13** |
| Sadness – hunger | 3.04 (1.86) | 3.75 (2.12) | 2.51** |
| Sadness – thirst | 2.35 (1.73) | 3.54 (2.08) | 4.04*** |
| Sadness – ache pain | 3.01 (1.83) | 3.87 (1.93) | 3.06** |
| Sadness – physical fatigue | 3.60 (1.85) | 4.54 (2.01) | 3.33** |
| Sadness – shortness of breath | 2.42 (1.74) | 3.16 (2.09) | 2.49* |
| Sadness – muscle cramp/tension | 2.78 (1.81) | 3.79 (1.95) | 3.66*** |
| Sadness – numbness | 2.85 (1.88) | 4.30 (2.25) | 4.54*** |
| Sadness – skin tingling/irritation | 2.33 (1.84) | 3.43 (2.19) | 3.53** |
| Sadness – racing heart | 2.22 (1.66) | 3.49 (2.29) | 4.05*** |
| Disgust - hot | 2.56 (1.87) | 3.55 (2.31) | 3.07** |
| Disgust – cold | 1.69 (1.24) | 2.28 (1.70) | 2.52* |
| Disgust – nausea | 3.86 (2.10) | 4.78 (2.09) | 3.90** |
| Disgust – hunger | 1.75 (1.37) | 2.73 (1.94) | 3.71*** |
| Disgust – thirst | 1.38 (.84) | 2.30 (1.64) | 4.3*** |
| Disgust – ache pain | 1.52 (1.06) | 2.33 (1.70) | 3.55** |
| Disgust – physical fatigue | 1.50 (1.01) | 2.37 (1.57) | 4.15*** |
| Disgust – shortness of breath | 1.58 (1.28) | 2.55 (1.79) | 3.99*** |
| Disgust – muscle cramp/tension | 1.37 (.86) | 2.30 (1.68) | 4.27*** |
| Disgust – numbness | 1.53 (1.28) | 2.55 (1.91) | 3.97*** |
| Disgust – skin tingling/irritation | 1.99 (1.40) | 3.13 (2.05) | 4.09*** |
| Disgust – racing heart | 1.82 (1.34) | 2.55 (1.60) | 3.23** |
| Anger - hot | 4.10 (1.90) | 4.61 (2.15) | 1.70 |
| Anger – cold | 2.09 (1.52) | 2.90 (1.93) | 2.97** |
| Anger – nausea | 2.27 (1.60) | 3.82 (1.97) | 5.60*** |
| Anger – hunger | 2.71 (2.08) | 3.36 (2.32) | 1.90 |
| Anger – thirst | 1.99 (1.51) | 2.58 (1.89) | 2.23* |
| Anger – ache pain | 2.07 (1.56) | 3.33 (2.02) | 4.47*** |
| Anger – physical fatigue | 2.20 (1.62) | 3.67 (1.27) | 4.73*** |
| Anger – shortness of breath | 2.23 (1.59) | 3.18 (2.04) | 3.34** |
| Anger – muscle cramp/tension | 2.44 (1.69) | 3.55 (2.13) | 3.71*** |
| Anger – numbness | 1.71 (1.27) | 2.94 (2.10) | 4.40*** |
| Anger – skin tingling/irritation | 2.54 (1.80) | 3.78 (2.18) | 4.02*** |
| Anger – racing heart | 3.57 (2.12) | 4.61 (2.26) | 3.19** |
| Fear - hot | 2.18 (1.71) | 2.33 (1.77) | .533 |
| Fear – cold | 2.74 (1.75) | 3.72 (2.05) | 3.56*** |
| Fear – nausea | 3.70 (1.93) | 4.54 (2.04) | 2.79** |
| Fear – hunger | 1.71 (1.30) | 2.61 (2.13) | 3.25** |
| Fear – thirst | 1.77 (1.32) | 2.69 (1.93) | 3.50** |
| Fear – ache pain | 2.04 (1.47) | 2.87 (1.90) | 3.13** |
| Fear – physical fatigue | 1.89 (1.47) | 2.72 (2.03) | 2.94** |
| Fear – shortness of breath | 3.57 (2.05) | 4.30 (2.23) | 2.36* |
| Fear – muscle cramp/tension | 2.02 (1.55) | 2.96 (2.13) | 3.26** |
| Fear – numbness | 2.08 (1.63) | 3.21 (2.18) | 3.73*** |
| Fear – skin tingling/irritation | 2.19 (1.68) | 3.15 (2.15) | 3.19** |
| Fear – racing heart | 4.42 (2.00) | 5.25 (1.99) | 2.84** |
| Surprise - hot | 2.15 (1.73) | 2.46 (1.80) | 1.15 |
| Surprise – cold | 1.51 (1.12) | 1.91 (1.29) | 2.14* |
| Surprise – nausea | 1.75 (1.31) | 2.87 (1.92) | 4.27*** |
| Surprise – hunger | 1.24 (.65) | 1.73 (1.16) | 3.22** |
| Surprise – thirst | 1.31 (.91) | 1.79 (1.25) | 2.80* |
| Surprise – ache pain | 1.35 (.81) | 1.85 (1.22) | 3.02** |
| Surprise – physical fatigue | 1.30 (.83) | 1.76 (1.34) | 2.56* |
| Surprise – shortness of breath | 2.23 (1.67) | 3.03 (1.96) | 2.86** |
| Surprise – muscle cramp/tension | 1.48 (1.13) | 2.28 (1.68) | 3.55** |
| Surprise – numbness | 1.56 (1.11) | 2.21 (1.59) | 3.03 |
| Surprise – skin tingling/irritation | 1.63 (1.21) | 2.42 (1.74) | 3.32* |
| Surprise – racing heart | 3.49 (2.12) | 4.13 (2.21) | 2.03 |
